# Correlation between maximum heart distance and thoracic diameter changes and diaphragmatic descent in left-sided breast cancer patients during deep inspiration breath-hold (DIBH)

**DOI:** 10.2478/raon-2023-0053

**Published:** 2023-11-30

**Authors:** He-Gou Wu, Guang-Wei Zhang, Jian-Feng Liu, Jun-Guo Yang, Xiao-Hui Su

**Affiliations:** Department of Radiation Oncology, Shenzhen People's Hospital, The Second Clinical Medical College of Jinan University, Shenzhen, Guangdong, People's Republic of China

**Keywords:** left-sided breast cancer, maximum heart distance, thoracic diameter, diaphragmatic descent, deep inspiration breath-hold

## Abstract

**Background:**

Cardioprotection is valued in radiotherapy for patients with left-sided breast cancer. Deep inspiration breath-hold (DIBH) technique can achieve cardioprotection well. However, during DIBH, the extent to which the heart enters the radiation field is affected by the movement of the thorax and diaphragm. The aim of this study was to analyze the correlation between the maximum distance of the heart entering the field (maximum heart distance, MHD) and thoracic diameter changes and diaphragmatic descent in left-sided breast cancer patients during DIBH.

**Patients and methods:**

Ninety-eight patients with left-sided breast cancer were included in this retrospective study. They performed simulation in Sentinel-guided DIBH, and two sets of CT images were collected under both free breathing (FB) and DIBH, and diaphragm positions, anteroposterior thoracic diameter (ATD), transverse thoracic diameter (TTD), gating window level (GWL), and MHD were measured, and the change (Δ) of each parameter in DIBH relative to that in FB were calculated. Pearson or Spearman test were used to analyze the correlation between ΔMHD and the changes in other parameters.

**Results:**

For all patients with DIBH, the average of ΔMHD was −8.3 mm, and the average of ΔATD and ΔTTD were 11.0 and 8.6 mm, and the median of both left diaphragmatic descent (LDD) and right diaphragmatic descent (RDD) were 35.0 mm, and the median of GWL was 11.1 mm. The correlation coefficients between MHD decrease (ΔMHD) and LDD, RDD, and ΔTTD were −0.430 (p = 0.000), −0.592 (p = 0.000) and 0.208 (p = 0.040), respectively, but not significantly correlated with ΔATD or GWL.

**Conclusions:**

The MHD decrease showed a moderate correlation with diaphragmatic descent In Sentinel-guided DIBH for patients with left-sided breast cancer, while there was a weak or no correlation with thoracic diameter changes or GWL. Abdominal breathing can lower diaphragm more and may be more beneficial to the heart stay away from tangential field.

## Background

In radiotherapy for left-sided breast cancer, it is necessary to face issues such as heart protection, lung protection, and the influence of respiratory motion, *etc*. As an important organ adjacent to the breast, the heart has received special attention in radiotherapy for left-sided breast cancer. Studies have shown that the increase in the dose to the heart has a linear relationship with the increase in major coronary events^1^. Therefore, in clinical practice of radiotherapy for left-sided breast cancer, cardioprotection has increasingly become the focus of attention. Deep inspiration breath-hold (DIBH) technology can increase the distance between the heart and the chest wall, keep the heart away from the center of the radiation field, and reduce the volume of the heart entering the radiation field^2^, thereby achieving the purpose of protecting the heart. Many studies have shown that the use of DIBH technology in radiotherapy for left-sided breast cancer can effectively reduce the dose to the heart^3,4,5,6,7^. Other studies have shown a strong correlation between the maximum distance of the heart into the tangential field (maximum heart distance, MHD) and cardiac dose reduction^8,9,10^. Therefore, to a certain extent, MHD can reflect the level of cardiac exposure, and can even be used as a predictor of the average cardiac exposure dose^10^, so it has a positive effect on clinical work to find out which factors affect MHD. However, there are few reports on the relationship between the extent of the heart entering the radiation field (MHD size) and the movement of the thorax and diaphragm during DIBH. In this article, we will study the correlation between MHD change and thoracic diameter changes and diaphragm descent, and reveal the main factors affecting MHD, so as to provide reference for the clinical practice of radiotherapy in DIBH for left-sided breast cancer.

## Patients and methods

Ninety-eight patients with left-sided breast cancer (age range 26 to 64 years, median age 44.0 years) were included in this retrospective study. They received radiotherapy in DIBH in our center from January 2020 to May 2022. This study was performed in line with the principles of the Declaration of Helsinki. Approval was granted by the Ethics Committee of Shenzhen People's Hospital (Date 2021-2-24/No LL-KY-2021047). Written informed consent was waved because this was a retrospective study.

### CT simulation

All patients in this study were immobilized by vacuum bags in supine position with both arms lifted up. CT simulation was performed under the guiding of Sentinel which is a CT-end of the Catalyst^TM^ system--an optical surface guided system (C-RAD AB, Uppsala, Sweden)^11^. Before CT simulation, all patients underwent Sentinel-guided DIBH breathing training under the guidance of doctors. No uniform requirement was made for breathing pattern during DIBH, and each patient chose his most comfortable breathing pattern, which can be thoracic breathing, abdominal breathing or mixed breathing. The cone-beam CT (CBCT) scan before delivered usually takes more than 40 seconds, in order to meet the scanning duration of CBCT, the patient's DIBH duration was required to be ≥ 40 s. Sentinel detects patient's breathing through a gating point, which is a region with a diameter of 4 cm located on the patient's skin surface, and Sentinel monitors the spatial position of the gating point by emitting a laser (wavelength: 635–690 nm, frequency: 15 Hz) to monitor the breath-holding state. In this study, the gating point was placed at 1–2 cm superior to the xiphoid process. Sentinel uses the gating window to set the allowable range of chest rise and fall during breath holding, the lower limit of the allowable range is “LOW”, the upper limit of the allowable range is “HIGH”, and the distance between “HIGH” and “LOW” is the width of the gating window. In this study, the width of the gating window was set to 3 mm, and the gating window level (distance from Baseline to “LOW”, GWL) is not uniformly required, but the GWL should exceed the amplitude of free breathing (FB). Sentinel is equipped with goggles, which provide visual feedback for patients during breathing training or CT scanning. Two sets of CT images (CTfb, CTdibh) were acquired for each patient using SOMATOM Definition AS (SIEMENS Healthcare, Germany) under FB and DIBH. Scanning parameters were 120 KV, 140 mAs (CARE Dose4D was selected), and the slice thickness was 5 mm.

### Patient coordinate system and respiratory characteristic parameters measurement

**FIGURE 1.**
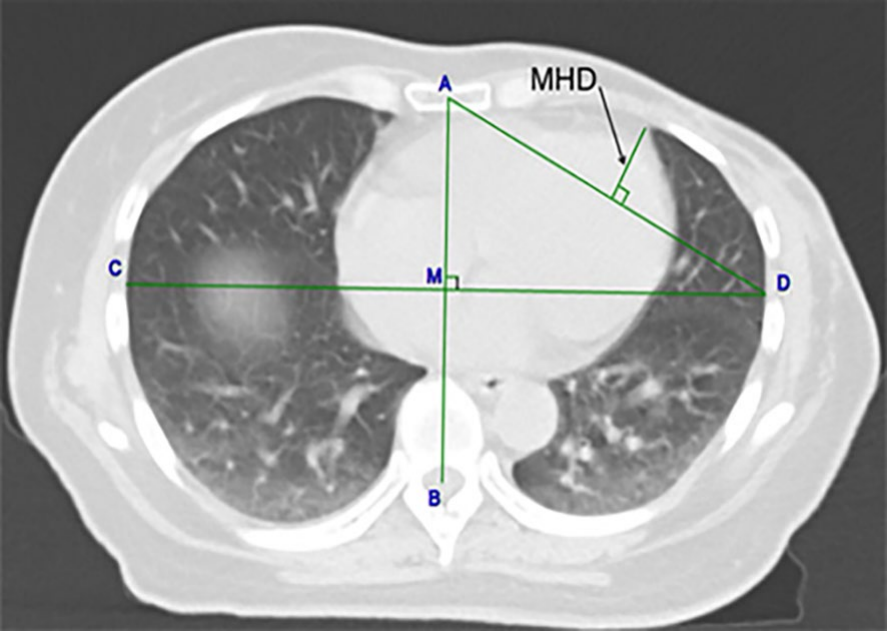
Parameter measurement diagram. For each patient, at the height of the vertebral body corresponding to the right diaphragm top during free breathing, anteroposterior thoracic diameter (ATD), transverse thoracic diameter (TTD) and maximum heart distance (MHD) were measured on free breathing CT images and deep inspiration breath-hold CT images respectively. Point A at the center of the sternum, and point B at the center of the medulla, the line AB was constructed as the ATD. Through the center of the line AB at M, a second line was constructed perpendicular to AB reaching the lateral edge of the lung at C and D, and the line CD was the TTD. The line AD approximates the direction of the tangential field, and the maximum distance from the left edge of the heart to AD is taken as the MHD.

In order to evaluate the size of the heart entering the radiation field in patients with DIBH, we refer to the measurement method of Kenneth Wikström^12^, and take the maximum distance of the heart edge into the simulated tangential field (MHD) as an evaluation parameter. The position of MHD is usually at the level of the right diaphragmatic top, so we chose the level of the right diaphragmatic top in FB as the location for the simulated measurement. On the CTfb and CTdibh, we measured the thoracic diameters and MHD in the FB and DIBH, respectively (Figure 1). DICOM image viewing software CARESTREAM Vue PACS (Version 12.2.0.0314, Carestream Health Inc., USA) were used to measure the parameters. The window width during measurement is 1600, and the window level is −599. First, on the CTfb images, we construct a line from the center of the sternum (A) to the center of the spinal cord cavity (B), which is the anteroposterior thoracic diameter (ATDfb). Draw a perpendicular to AB at the midpoint of AB, and the vertical line reaches the right edge of the lung (C) and the left edge of the lung (D). CD is the transverse thoracic diameter (TTDfb). Connect the two points A and D, and measure the maximum distance from the edge of the heart to AD as the maximum heart distance (MHDfb). Second, using the above method, on the CTdibh image, which is equivalent to the same level of vertebral body height corresponding to the right diaphragmatic top in FB, the anteroposterior thoracic diameter (ATDdibh), transverse thoracic diameter (TTDdibh), and maximum heart distance (MHDdibh) were measured.

### Respiratory characteristic parameters calculation

The change of each parameter reflected the change of DIBH state relative to FB state. Accordingly, this study uses the difference of parameters in the two states for evaluation. For each patient, the parameter difference was calculated as follows: First, ATDfb was subtracted from ATDdibh to obtain the increase in ATD (ΔATD) in DIBH. Second, subtract TTDfb from TTDdibh to obtain the increase in TTD (ΔTTD) in DIBH. Third, MHDfb was subtracted from MHDdibh to obtain the reduction in MHD in DIBH (ΔMHD, It's usually a negative number). Fourth, measure the downward displacement of the diaphragm in DIBH: left diaphragmatic descent (LDD), right diaphragmatic descent (RDD). On CT images of FB and DIBH, take the position of the vertebral body corresponding to the level of the diaphragm dome in FB as the reference, and the vertical descending distance of the diaphragm dome in DIBH relative to the reference is LDD or RDD. Fifth, in Sentinel gating window, the “LOW” minus the “Baseline” is used as the gating window level (GWL).

### Dosimetric parameters

According to the RTOG guidelines, target volumes and organs at risk (OARs) were contoured on Eclipse (version 13.6, Varian Medical Systems, Palo Alto, CA). 3D conformal radiotherapy (3DCRT) plans were generated on both the DIBH and FB simulation CT images for dosimetric analysis. Each patient prescribed 40.5 Gy in 15 fractions for whole breast and chest wall. Four dosimetric parameters were obtained, including mean heart dose (Dmean), heart volume in field (HVIF), which is the heart volume contained in the 50% isodose line, percentage volumes of the heart receiving doses ≥ 10Gy (V10), and percentage volumes of the heart receiving doses ≥ 5Gy (V5). Then, subtract the value of a parameter in FB from the value of this parameter in DIBH to obtain each parameter difference (ΔDmean, ΔHVIF, ΔV10, ΔV5).

### Data analysis

This study analyzed the correlation between change in MHD and changes in other parameters. After calculating geometric parameters (ΔMHD, ΔATD, ΔTTD, RDD, LDD, GWL) and dosimetric parameters (ΔDmean, ΔHVIF, ΔV10, ΔV5), statistical analysis was performed using SPSS 25 (IBM Corp.). For normal data, the mean (± standard deviation) was used to describe, and Pearson correlation coefficient test was used for correlation analysis; for non-normal data, median (range) was used to describe and Spearman correlation coefficient test was used for correlation analysis. P < 0.05 means the difference is statistically significant.

## Result

### Respiratory characteristic parameters and GWL

The measured values and calculation results of each respiratory characteristic parameter are shown in Table 1. Among the 98 patients, 91 patients (92.9%) had MHD reduction to varying degrees, the largest reduction in MHD was −22.2 mm (ΔMHD), and 7 patients (7.1%) had increased MHD in DIBH (ΔMHD were 3.75, 1.29, 1.26, 0.93, 0.68, 0.49, 0.10 mm, respectively). Of these patients with increased MHD in DIBH, in addition to 2 who had pleural effusion and prosthesis implantation respectively, in the other 5 patients with increased MHD, the largest increase in MHD was 1.26 mm. Moreover, during DIBH, one patient had no change in left diaphragm (LDD 0 mm), and there was a patient who had no descending in the right diaphragm (RDD 0mm), and another one who had a decrease in the TTD (ΔTTD −2 mm).

**TABLE 1.** The values of respiratory characteristic parameters in free breathing (FB) and/or deep inspiration breath holding (DIBH), and the differences in the values of some respiratory characteristic parameters between DIBH and FB (N = 98)

**Parameters**	**FB**	**DIBH**	**Δ**
**MHD (mm)**	26.8 (±5.1)	18.4(±7.5)	−8.3 (±5.9)
**ATD (mm)**	127. 0(±11.6)	139.0(±11.6)	11.0 (±3.8)
**TTD (mm)**	227.0 (±10.6)	235.6(±10.4)	8.6 (±4.4)
**RDD (mm)**		35.0(0.0 – 60.0)	
**LDD (mm)**		35.0(0.0 – 55.0)	
**GWL (mm)**		11.1(4.6 – 20.7)	

ATD = anteroposterior thoracic diameter; GWL = gating-window level; LDD = left diaphragmatic descent; MHD = maximum heart distance; RDD = right diaphragmatic descent; TTD = transverse thoracic diameter; Δ = differences between DIBH and FB

### Correlation between ΔMHD and thoracic diameter changes and diaphragmatic descent

**FIGURE 2.**
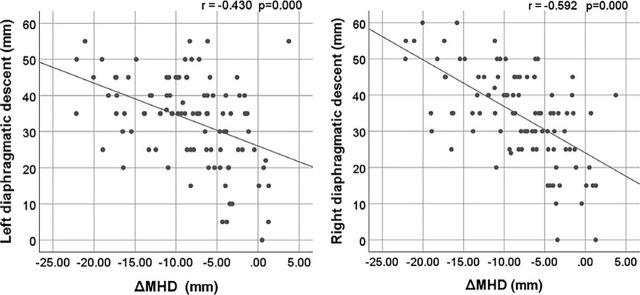
Correlation between ΔMHD and left diaphragmatic descent, right diaphragmatic descent [r: Spearman rank correlation coefficient; p: p-value (2-tailed)]. ΔMHD = maximum heart distance in deep inspiration breath-hold minus maximum heart distance in free breathing

In this study, ΔMHD showed a moderate correlation with RDD and LDD (Figure 2), the r-values were −0.592 (p = 0.000), −0.430 (p = 0.000). ΔMHD was weakly correlated with ΔTTD, the r-value was 0.208 (p = 0.040), while it was no significant correlation with ΔATD. Since there was only a weak correlation between ΔMHD and ΔTTD, we grouped ΔATD and ΔTTD according to the mean. For all patients, the mean of ΔATD was 11.0 mm, and the mean of ΔTTD was 8.6 mm. Patients were divided into high and low groups according to the means, which were ΔATDhigh (ΔATD > = 11.0 mm, 52 patients), ΔATDlow (ΔATD < 11.0 mm, 46 patients), ΔTTDhigh (ΔTTD > = 8.6 mm, 47 patients), ΔTTDlow (ΔTTD < 8.6 mm, 51 patients), the mean of parameters in each group are shown in Table 2. Statistical analysis showed that there was a statistical difference in ΔMHD between the ΔATDhigh and ΔATDlow groups (t = 2.179, p = 0.032), and that ΔMHD was correlated with ΔATD in the ΔATDlow group (r = −0.372, p = 0.011), but not in the ΔATDhigh group. Correspondingly, the means of ΔMHD in ΔTTDhigh and ΔTTDlow were also statistically different (Z = 1.966, p = 0.049), while ΔMHD was not correlated with ΔTTD in the two groups.

**TABLE 2.** Comparison of the mean of parameters in ΔATDlow (n = 46) and ΔATDhigh (n = 52) and in ΔTTDlow (n = 51) and ΔTTDhigh (n = 47)

	**LDD**	**RDD**	**ΔATD**	**ΔTTD**	**ΔMHD**
**ΔATDlow (mm)**	32.2	34.0	7.7	7.5	−7.0
**ΔATDhigh (mm)**	34.2	35.4	13.8	9.6	−9.5
**Increase of ΔATDhigh (mm)**	2.0	1.4	6.1	2.1	−2.5
**Increase of ΔATDhigh (%)**	6.2%	4.1%	78.9%	27.9%	36.5%
**ΔTTDlow(mm)**	35.3	37.1	10.5	5.4	−9.7
**ΔTTDhigh (mm)**	31.1	32.2	11.4	12.0	−6.9
**Increase of ΔTTDhigh (mm)**	−4.2	−4.9	0.8	6.6	2.8
**Increase of ΔTTDhigh (%)**	−12.0%	−13.2%	7.9%	122.2%	−29.3%

ATD = anteroposterior thoracic diameter: ΔATDlow = group with ΔATD < 11 mm; ΔATDhigh = group with ΔATD > =11 mm; ΔTTDlow = group with ΔTTD < 8.6 mm; TTD = transverse thoracic diameter; ΔTTDhigh = group with ΔTTD > = 8.6 mm

### Correlation between MHD and GWL

Using Sentinel to guide DIBH in patients with left-sided breast cancer, the degree of chest expansion is reflected by GWL. In this study, the patient's breathing pattern was not specified, and each patient chose a comfortable breathing pattern to achieve DIBH. As a result, the GWL of the patients varied relatively widely, ranging from 4.6 mm to 20.7 mm. However, it was found that there was no significant correlation between ΔMHD and GWL. Furthermore, the patients were divided into three groups according to GWL: Group 1: < 9 mm (24 patients), Group 2: ≥ 9 mm and < 15 mm (63 patients), Group 3: ≥ 15 mm (11 patients), no significant correlation between GWL and ΔMHD was found in each group, and no statistical difference was found in the ΔMHD among the three groups.

### Dosimetric parameters and their correlation with geometric parameters

In 95 of the 98 patients (96.9%), Dmean, HVIF, V10, and V5 were all reduced to varying degrees in DIBH compared with FB, with a maximum reduction of 590.1 cGy, 15.7%, 19.7%, and 21.7%, respectively, and the median of Dmean, ΔHVIF, ΔV10, and ΔV5 were: −176.1 cGy, −3.981%, −4.764%, −5.444%, respectively. Meanwhile, the dosimetric parameters showed moderate correlation with the geometric parameters ΔMHD, RDD, and LDD, as shown in Table 3.

**TABLE 3.** Correlation between dosimetric parameters and geometric parameters

		**ΔDmean**	**ΔHVIF**	**ΔV10**	**ΔV5**
**ΔMHD**	r	0.468	0.464	0.481	0.484
	p	< 0.001	< 0.001	< 0.001	< 0.001
**LDD**	r	− 0.340	− 0.365	− 0.382	− 0.373
	p	= 0.001	< 0.001	< 0.001	< 0.001
**RDD**	r	− 0.439	− 0.449	− 0.462	− 0.458
	p	< 0.001	< 0.001	< 0.001	< 0.001
**ΔATD**	r	0.007	0.044	0.026	0.008
	p	0.943	0.669	0.801	0.935
**ΔTTD**	r	0.012	0.055	0.062	0.065
	P	0.906	0.593	0.545	0.527

ATD = anteroposterior thoracic diameter; Dmean = heart mean dose; HVIF = heart volume in field, that's the heart volume within the 50% isodose line; LDD = left diaphragmatic descent; MHD = maximum heart distance; RDD = right diaphragmatic descent; TTD = transverse thoracic diameter; V5 = percentage volumes of heart receiving doses ≥ 5Gy; V10 = percentage volumes of heart receiving doses ≥ 10 Gy; Δ the value of a parameter in deep inspiration breath holding (DIBH) minus the value of this parameter in free breathing (FB)

## Discussion

In this article, the correlation between ΔMHD and the thoracic diameter changes and diaphragmatic descent was studied. Ninety-eight patients with left-sided breast cancer were simulated in Sentinel-guided DIBH. Their MHD, thoracic diameters, and diaphragmatic descent were measured on CTfb and CTdibh. It was found that the change of MHD were moderately correlated with the left and right diaphragmatic descent [−0.430 (p = 0.000), −0.592 (p = 0.000)], while the correlation with thoracic diameter changes was weak, only weakly correlated with ΔTTD (r = 0.208), and not correlated with ΔATD. This shows that in DIBH, the greater descending of the diaphragm, the smaller the MHD, and the change of thoracic diameters does not seem to have a significant effect on MHD. Meanwhile, it was also found that RDD and LDD were moderately correlated with ΔDmean, ΔHVIF, ΔV10 and ΔV5 through cardiac dose analysis (Table 3), which indicated that the greater the diaphragmatic descent, the greater the decrease in Dmean, HVIF, V10, V5. Several previous studies have shown that MHD is associated or strongly associated with cardiac dose^8,9,10,13,14^. Similar results were obtained in this study, ΔMHD and ΔDmean showed moderate correlation (Table 3). However, thoracic diameter changes were only weakly or not linearly associated with MHD, and were not associated with heart dosimetric parameters. For those patients with large thoracic diameter changes, is there a correlation between ΔMHD and ΔATD or ΔTTD? The result was also negative. In this study, 52 patients had ΔATD greater than the mean (11.0 mm), and 47 patients had ΔTTD greater than the mean (8.6 mm). However, there was still no significant correlation between their ΔMHD and ΔATD or ΔTTD. This may be because the expansion of the chest wall is anisotropic, it is difficult to expand uniformly in all directions, and it is difficult to show a linear change with the MHD even in the case of a greater degree of the chest wall expansion. In addition, in the case of further inspiration, the change of diaphragm position was also greater, which may also have influenced the correlation of MHD with chest wall expansion. The reduction of MHD by DIBH is usually caused by the combination of two factors. One is that diaphragm descending elongates the heart in the craniocaudal direction, reducing the transverse diameter of heart, thus keeping the heart away from the radiation field, and the other is that the lungs are more inflated in DIBH, keeping the chest wall away from the heart. The reason for the results of this study may be that the former has a more lasting effect on reducing MHD than the latter, because the movement range of the diaphragm was larger than that of the chest wall (Table 1). In DIBH, after the chest wall has expanded to a certain extent, the diaphragm can still descend further and continue to have an effect on the reduction of MHD, so that a linear change may be formed between the diaphragmatic descent and the reduction of MHD. In addition, as mentioned earlier, the expansion of the chest cavity is anisotropic, which makes it difficult to form a relatively regular change in the distance between the heart and the chest wall, so the reduction of MHD is not easy to show a linear relationship with the thoracic diameters.

Although there was weak or no linear correlation between MHD and thoracic diameters, different thoracic diameters would result in statistical differences in ΔMHD. According to the thoracic diameter grouping data, it was found that there were statistical differences in ΔMHD between the thoracic diameters high and low groups. MHD was smaller in ΔATDhigh group than in ΔATDlow group (ΔMHD mean −9.5 *vs* −7.0 mm), which meant that the hearts of patients in ΔATDhigh group were farther away from the tangent field, while the opposite was true in the grouping of ΔTTD, where MHD is greater in ΔTTDhigh than in ΔTTDlow (ΔMHD mean −6.9 *vs* −9.7 mm), thus it can be seen that the ΔMHD of patients with ΔATD greater than the mean benefited from the increase of ATD, but this was not the case for patients with ΔTTD greater than the mean, and the increase in TTD did not contribute further to the heart moving away from the radiation field. Further comparison of the diaphragmatic descent between the high and low groups, it was found that the diaphragmatic descent of ΔATDhigh group was slightly greater than that of the ΔATDlow group (LDD 34.2 *vs* 32.2 mm, RDD 35.4 *vs* 34.0 mm), but that of the ΔTTDhigh group was smaller than that of the ΔTTDlow group (LDD 31.1 *vs* 35.3 mm, RDD 32.2 *vs* 37.1 mm), indicating that after the TTD increases to a certain extent, a continued increase in TTD will lead to the diaphragm rising, which is not conducive to the heart away from the radiation field. Therefore, during DIBH training, it is necessary to choose a relatively large but not excessive inspiratory volume. In this way, the thoracic diameters and the diaphragmatic descent increase, which will help to keep the heart away from the field. When inappropriate inspiratory volume or inspiratory pattern leads to excessive expansion of the chest, it will negatively affect the heart away from the field.

Although the majority of patients had varying degrees of reduction in MHD in DIBH, but in this study, there were still 7 patients (7.1%) with increased MHD (range 0.10 to 3.75 mm), obviously, the ΔMHD of these patients did not benefit from DIBH technique. In 4 of the 7 patients, there were several factors that could affect DIBH, including pleural effusion, prosthesis implantation, left diaphragm not descending during DIBH, and the right diaphragm did not descend during DIBH, while no obvious factors affecting DIBH were found in the other 3 patients. In addition, one patient's right diaphragm did not descend (RDD 0mm) in DIBH, but her TTD increased significantly (ΔTTD 14.5 mm), and left diaphragm descended (LDD 10.0 mm), and her MHD decreased accordingly (ΔMHD −3.44 mm). Similarly, another patient's TTD decreased rather than increased in DIBH (ΔTTD −2 mm) and her ATD also increased less (ΔATD 4 mm), but her left and right diaphragms descended significantly (LDD 45 mm, RDD 50 mm), and MHD was also reduced accordingly (ΔMHD −5.86 mm). It can be seen from the above that in DIBH, there are various factors affecting the reduction of MHD, including local lesions, prosthesis implantation, respiratory patterns, *etc*. Tanguturi *et al.* found that the mean heart dose was increased in 14 of 146 patients (10%) in DIBH^15^. It shows that these patients did not benefit from DIBH technology. Dell’Oro *et al.* observed that in DIBH, 1 of 20 patients (5%) had increased MHD^16^, which was similar to what was observed in this study. DIBH reduces MHD by keeping the heart away from the radiation field, but in order to achieve effective DIBH, in addition to adequate breathing training, it is necessary to consider possible affecting factors and to screen patients by optimizing the conditions.

Different breathing patterns have different effects on the geometric changes of thoracic anatomy, which may also affect the extent to which the heart enters the field. Breathing patterns are generally divided into thoracic breathing, abdominal breathing and mixed breathing. According to the study of Kimiko Hirata *et al.*, In DIBH-guided radiotherapy for left-sided breast cancer, there was no statistical difference between thoracic breathing and abdominal breathing on the heart doses and the displacement between the heart and the target^17^. Zhao *et al.* evaluated T-DIBH and A-DIBH with three-dimensional conformal radiotherapy (3DCRT) and intensity-modulated radiation therapy (IMRT) in 22 patients^18^, their conclusion is that abdominal breathing significantly reduces the heart doses in both 3DCRT and IMRT. In this study, when the patient performed DIBH, the breathing patterns was not limited, and the patient may perform any of the three breathing patterns, but it can be inferred from the moderate correlation between the ΔMHD and the diaphragmatic descent that abdominal breathing may be more beneficial for keeping the heart away from the field.

When Sentinel guides DIBH of left-sided breast cancer patients, Sentinel relies on the gating window level (GWL) to monitor chest wall, which ensures the reproducibility of the chest in DIBH^19^. However, we found that there was no significant correlation between ΔMHD and GWL, and there was no significant difference in ΔMHD among the three GWL groups (< 9 mm, ≥ 9 mm and < 15 mm, ≥15 mm). In a previous study, Leigh Conroy *et al.* also found no correlation between the breath-hold level and left anterior descending artery chest wall separation^20^. Tuomas Koivumäki divided the GWL into two groups, low (7–12 mm, 8 patients) and high (13–20 mm, 7 patients), and the results showed that the heart position in the low GWL group had greater variability during treatment^21^. Therefore, even though the GWL has little correlation with the extent to which the heart enters the radiation field, it is still recommended to optimize the GWL to make the patient's DIBH state more stable.

A limitation of this study is that the difference in MHD between thoracic breathing and abdominal breathing was not analyzed because patients’ breathing pattern could not be assigned due to the retrospective data analysis.

Overall, the MHD decrease showed a moderate correlation with the diaphragmatic descent In Sentinel-guided DIBH for patients with left-sided breast cancer, while there was a weak or no correlation with thoracic diameter changes or GWL. Abdominal breathing can lower the diaphragm more and may be more beneficial to the heart stay away from the tangential field in clinical practice.
